# Book Review

**Published:** 1950-10

**Authors:** 


*We desire to acknowledge with thanks the receipt of the following books; too late for the inclusion of reviews in this issue.*

**Cerebral Angiography.** By P. ALMEIDA LIMA. Pp. xiv, 221. Illustrated. London: Oxford University Press, Geoffrey Cumberlege. 1950. Price 45s.

**Biological Standardization.** By J. H. BURN, D. J. FINNEY and L. G. Goodwin. Second Edition. Pp. x, 440. Illustrated. London: Oxford University Press, Geoffrey Cumberlege. 1950. Price 35s.

**Medical Annual, 1950.** Edited by Sir HENRY TIDY, K.B.E., M.D., F.R.C.P., and A. RENDLE SHORT, M.D., B.S., B.Sc., F.R.C.S. Pp. 440. Illustrated. Bristol: John Wright & Sons Ltd. 1950. Price 25s.

**Eye Surgery.** By H. B. STALLARD, M.B.E., M.D., F.R.C.S. Second Edition. Pp. xvi, 683. Illustrated. Bristol: John Wright & Sons Ltd. 1950. Price 52s. 6d.

**Minor Surgery.** Edited by Sir HENEAGE OGILVIE, K.B.E., D.M., M.Ch., F.R.C.S. and W. A. R. THOMSON, M.D. Second Edition. Pp. xiv, 92. Illustrated. London: *The Practitioner*. 1949. Price 14s.

[*continued on page 127*

### REVIEWS OF BOOKS—(*continued from page 123.*)

**Sir Benjamin Ward Richardson.** By Sir Arthur Salusbury MacNalty, K.C.B., M.A., M.D., F.R.C.P., F.R.C.S. Pp. vii, 92. London: Harvey & Blythe Ltd. 1950. Price 7s. 6d.

**Infant Nutrition.** By F. W. Clements, M.D., D.P.H., D.T.M. Pp. 254. Illustrated. Bristol: John Wright & Sons Ltd. 1949. Price 21s.

**Clinical Examination of Patients.** By John Forbes, M.D., M.R.C.P. and W. N. Mann, M.D., F.R.C.P. Pp. x, 323. Illustrated. London: Edward Arnold & Co. 1950. Price 18s.

